# Physical distress is associated with cardiovascular events in a high risk population of elderly men

**DOI:** 10.1186/1471-2261-9-14

**Published:** 2009-03-30

**Authors:** Gunnar Einvik, Øivind Ekeberg, Tor O Klemsdal, Leiv Sandvik, Elsa M Hjerkinn

**Affiliations:** 1 Division of Medicine, Akershus University Hospital, Lørenskog, Norway; 2 Department of Behavioural Science, University of Oslo, Oslo, Norway; 3 Department of Acute Medicine, Oslo University Hospital Ullevål, Oslo, Norway; 4 Department of Preventive Cardiology, Oslo University Hospital Ullevål, Oslo, Norway; 5 Centre for Clinical Research, Oslo University Hospital Ullevål, Oslo, Norway; 6 Department of Cardiology, Oslo University Hospital Ullevål, Oslo, Norway

## Abstract

**Background:**

Self-reported health perceptions such as physical distress and quality of life are suggested independent predictors of mortality and morbidity in patients with established cardiovascular disease. This study examined the associations between these factors and three years incidence of cardiovascular events in a population of elderly men with long term hyperlipidemia.

**Methods:**

We studied observational data in a cohort of 433 men aged 64–76 years from a prospective, 2 × 2 factorial designed, three-year interventional trial. Information of classical risk factors was obtained and the following questionnaires were administered at baseline: Hospital Anxiety and Depression Scale, Physical Symptom Distress Index and Life Satisfaction Index. The occurrence of cardiovascular death, myocardial infarction, cerebrovascular incidences and peripheral arterial disease were registered throughout the study period. Continuous data with skewed distribution was split into tertiles. Hazard ratios (HR) were calculated from Cox regression analyses to assess the associations between physical distress, quality of life and cardiovascular events.

**Results:**

After three years, 49 cardiovascular events were registered, with similar incidence among subjects with and without established cardiovascular disease. In multivariate analyses adjusted for age, smoking, systolic blood pressure, serum glucose, HADS-anxiety and treatment-intervention, physical distress was positively associated (HR 3.1, 95% CI 1.2 – 7.9 for 3^rd ^versus 1^st ^tertile) and quality of life negatively associated (HR 2.6, 95% CI 1.1–5.8 for 3^rd ^versus 1^st ^tertile) with cardiovascular events. The association remained statistically significant only for physical distress (hazard ratio 2.8 95% CI 1.2 – 6.8, p < 0.05) when both variables were evaluated in the same model.

**Conclusion:**

Physical distress, but not quality of life, was independently associated with increased risk of cardiovascular events in an observational study of elderly men predominantly without established cardiovascular disease.

**Trial Registration:**

Trial registration: NCT00764010

## Background

Several psychosocial factors are shown to have independent adverse effects on cardiovascular mortality and morbidity. These include major depression [1], anxiety symptoms [2], type D personality (a stable tendency of generally negative affects and social inhibition) [3], low socio-economic status [4], lack of social support [5], stressful life events and job stress [6]. In addition, a positive psychological factor as optimism seems to have a protective effect [7]. In current European guidelines for cardiovascular prevention, evaluation of such factors is recommended in all patients, but is not a part of risk-score strategies [8].

The role of perceived distress from physical symptoms has been studied in various clinical materials. Epidemiological studies show an independent association between self-reporting symptoms as dyspnoea, cough and feeling cold and all-cause mortality [9]. In studies on patients with stable coronary heart disease, symptom score measured by the Seattle Angina Questionnaire is associated with increased disease-specific mortality when adjusted for established risk factors and objective measures of cardiac function [10]. Thus, measuring subjective patient perceptions of physical distress may yield information not revealed by objective examinations. This could be valuable when performing considerations of risk and primary preventive measures. We are not aware of studies examining self-reported physical distress in relation to novel cardiovascular events among patients at high risk of cardiovascular disease (CVD).

Quality of life (QOL) is a construct reflecting the patient's perception of several psychosocial factors. Such questionnaires are frequently applied as secondary outcomes in interventional studies, but have also been evaluated in prospective studies on cardiovascular health in recent years. Low QOL has been associated with increased mortality after cardiac surgery [11], percutaneous coronary intervention [12], acute myocardial infarction [13], and in stable coronary artery disease [10]. However, in healthy populations with risk factors of CVD, data is limited. Low well-being predicts stroke in elderly men with hypertension and hypercholesterolemia [14]. To our knowledge, the association between other cardiovascular events as death, myocardial infarction and revascularization procedures and low QOL has not been studied in a high risk population.

The aim of this study was to examine whether increased self-reported physical distress or low QOL is independently associated with three years incidence of cardiovascular mortality and morbidity among elderly men at high risk of CVD.

## Methods

### Study design and sample

**Figure 1:**
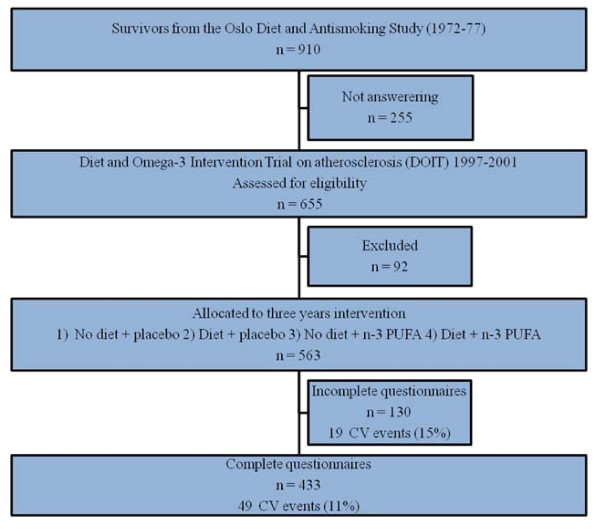
**Flow chart of the study population**. n-3 PUFA, n-3 polyunsaturated fatty acids; CV, cardiovascular.

The basis of recruitment in the present study, Diet and Omega-3 Intervention Trial on atherosclerosis (DOIT), were the 910 survivors from the original population of 1232 otherwise healthy men with hypercholesterolemia (>6.45 mmol/l) in the Oslo Diet and Antismoking Study, carried out from 1972–1977 [15] (Figure 1).

Altogether, 655 men aged 64–76 years attended a screening visit in 1997. Exclusion criteria in the DOIT were: Total cholesterol > 8 mmol/l, blood pressure levels > 170/100 mmHg, specific disease states thought to influence longevity or study compliance (cancer, end-stage renal failure, chronic alcoholism). A total of 77 were excluded prior to randomization and 15 were unwilling to participate.

The 563 participants were randomized to a 2 × 2 factorial designed three-year prospective study with n-3 polyunsaturated fatty acids and/or dietary counselling on progression of atherosclerosis measured by biochemical, functional and structural arterial wall properties. Further details of inclusion criteria, intervention and follow-up have previously been reported [16]. The present article reports observational data from a subgroup of participants in the DOIT with complete baseline data collection.

### Data collection

At randomisation, information about previous morbidity, medications and current smoking was registered. Data from clinical examinations were collected by one of the authors (EMH) and blood tests were drawn after overnight fasting under standard procedures.

The participants filled out the following questionnaires: Hospital Anxiety and Depression Scale (HADS) [17], Physical Symptom Distress Index (PSDI) [18] and Life Satisfaction Index (LSI) [19].

HADS is a 14-item questionnaire on symptoms of anxiety (HADS-A) and depression (HADS-D), each ranging from 0 (no problems) to 3 (maximum distress). Its reliability and validity as a screening instrument has been confirmed in cardiovascular patients [20]. PSDI quantifies physical distress using 13 questions on a scale ranging from 1 (not at all) to 5 (very much). It is a revised version of a questionnaire developed by The National Heart, Lung and Blood Institute for follow-up studies in hypertension [18]. Quality of life was measured by the 14-item LSI, using a scale of 1 (very satisfied) to 4 (very unsatisfied). PSDI and LSI have previously been used in Norwegian populations [21]. The individual items on PSDI and LSI are given in table 1.

**Table 1:** The individual items in the study questionnaires.

**Physical Symptom Distress Index**. "During the last four week, to what extent have you felt distressed by the following physical symptoms?"					
1.	Fatigue	14.8%	8.	Peripheral numbness	7.4%
2.	Dizziness	6.5%	9.	Ankle edema	6.2%
3.	Exanthema	7.1%	10.	Nightmares	3.4%
4.	Tiredness	21.8%	11.	Dry mouth	10.5%
5.	Slow heart rate	4.5%	12.	Loss of taste	2.3%
6.	Dyspnoea	16.5%	13.	Blurred vision	5.7%
7.	Cold hands	13.5%			
					
**Life Satisfaction Index**. "During the last four weeks, how satisfied have you been with the following factors in your life?"					
1.	Time spent with friends	5.1%	8.	Your marriage	3.2%
2.	Time spent with relatives	6.3%	9.	Your education	11.4%
3.	Your hobbies	9.9%	10.	The relation to your children	2.1%
4.	Participation in social activity in the community	16.6%	11.	The housing situation	1.4%
5.	Time spent with close family	6.0%	12.	Your economy	3.7%
6.	Your energy	17.9%	13.	Your standard of living	1.6%
7.	Your daily life	3.9%	14.	Your religion	10.0%

Participants with complete questionnaires (n = 433). Numbers referring to proportion reporting "some", "moderate", "much" or "very much" on PSDI and "unsatisfied" or "very unsatisfied" on LSI, respectively.

Questionnaires from individuals with one missing item on the HADS-subscales, up to two missing items on PSDI and four missing items on LSI were included in the analyses after simple imputation, as the missing items were estimated as the average from the other items.

The primary endpoint was a composite of cardiovascular death, myocardial infarction, percutaneous coronary intervention, coronary-artery bypass grafting, cerebral stroke, surgery on abdominal aortic aneurysm and revascularization procedures in peripheral arterial disease. The events were confirmed by medical records and data supplied from the death-cause register in Norway, and verified by an independent cardiologist.

The study was approved by the regional ethics committee, and all subjects gave their written informed consent prior to participation.

### Statistics

Continuous variables with normal distribution were standardized by dividing with its standard deviation (SD). Variables with skewed distributions were categorized in tertiles. The strength of bivariate correlation between continuous variables was assessed using Pearson's correlation analysis.

Univariate Cox proportional hazards regression analyses were used to estimate the associations between classical risk factors for CVD at baseline, physical distress, quality of life, and cardiovascular events. The hazard ratios were calculated as the increase in risk of event when the selected variable was increased by one SD or one tertile. Classical risk factors associated (p < 0.20) with the outcome in univariate analyses were entered in multivariate analyses, and those significantly (p < 0.05) associated with the outcome were kept into the model. Further, physical distress and quality of life were entered separately in the multivariate model. Finally, physical distress and quality of life were evaluated together in the same model.

## Results

A total of 433 subjects filled out all questionnaires at baseline, and were included in further analyses. Baseline characteristics of these participants are given in table 2. When applying standard European risk stratification (SCORE) [8], 369 subjects (85%) were considered to be at high risk or using antihypertensive medication or statins.

**Table 2:** Baseline demographics and clinical characteristics.

Age (years, range)	70 (64–75)
Previous cardiovascular disease (%)	26
Previous diabetes mellitus (%)	9
Treated hypertension (%)	26
Current smoking (%)	34
Systolic blood pressure (mmHg)	148 ± 18
Pulse rate (min^-1^)	68 ± 12
Body mass index (kg/m^2^)	26.8 ± 3.5
Total cholesterol (mmol/l)	6.3 ± 1.0
HDL-cholesterol (mmol/l)	1.4 ± 0.4
LDL-cholesterol (mmol/l)	4.1 ± 1.0
Triglycerides (mmol/l)	1.8 ± 0.9
Glucose (mmol/l)	6.0 ± 1.5
HADS-Anxiety	3.2 ± 2.6
HADS-Depression	3.4 ± 2.6
Physical Symptom Distress Index	18.8 ± 4.6
Life Satisfaction Index	23.7 ± 4.9

Participants with complete questionnaires (n = 433). Data are expressed as mean + SD, if not otherwise mentioned. Hospital Anxiety and Depression Scale 0–21, Physical Symptom Distress Index: Scale 13–65, Life Satisfaction Index: Scale 14–56.

HDL, High-density lipoprotein; LDL, Low-density lipoprotein; HADS, Hospital Anxiety and Depression Scale.

The 130 subjects with incomplete questionnaires had significantly higher systolic blood pressure (152 ± 18 vs. 148 ± 20, p = 0.036) and lower body mass index (25.9 ± 3.3 vs. 26.7 ± 3.5, p = 0.001), but showed no significant differences in other classical risk factors, anxiety or depression. The proportions of patients reporting distress or dissatisfaction on each item in PSDI and LSI are given in table 1.

There were 49 cardiovascular events among the 433 participants (11%), not significantly different from the 19 (15%) cardiovascular events among the 130 subjects without complete questionnaires.

The additional file 1 presents univariate associations between classical risk factors, psychosocial parameters and cardiovascular events. High physical distress and low QOL were significantly associated with the incidence of cardiovascular events.

In correlation analyses, PSDI and LSI were significantly correlated (p < 0.001) both with each other, and with HADS. The strongest correlation was between HADS-A and PSDI and HADS-D and LSI (r = 0.42, p < 0.001), the other r's ranging from 0.33 to 0.35.

Among the classical cardiovascular risk factors, previous cardiovascular disease, current smoking, level of serum glucose, diabetes, systolic blood pressure, LDL-cholesterol and in addition HADS-anxiety, were entered into the multivariate analyses together with the treatment modality. However, only level of serum glucose, systolic blood pressure, current smoking and HADS-anxiety were significantly associated with the outcome, and composed the multivariate model that our main co-factors PSDI and LSI were entered in.

When entering only PSDI in the model, subjects in the upper tertile PSDI had significantly increased hazard compared to subjects from the lower tertile (HR 3.1, 95% CI 1.2 – 7.9), while HADS-anxiety no longer was significantly associated. When entering only LSI, the association with the main outcome was somewhat stronger than in univariate analyses (HR 2.6, 95% CI 1.1–5.8). Finally, entering both co-factors at focus in the same multivariate model weakened the association between these and cardiovascular events, although still statistically significant for PSDI (additional file 1).

## Discussion

This study demonstrates that physical distress is independently associated with the three year incidence of cardiovascular events in high risk elderly men. We found no significant independent association between low QOL and cardiovascular events.

Our results support previous suggestions that an association between physical distress and incidence of cardiovascular events exists (10). Such a measure probably reflects two dimensions; The level of symptom burden, and the patient's perception of this.

Fatigue/tiredness, dyspnoea and peripheral cold were the most prevalent symptoms reported. These are general symptoms, but they are all associated with the cardiovascular system and circulation. One hypothesis is that such symptoms may represent atherosclerotic disease not yet manifested as end-organ damage or events, and is in accordance with previous data in healthy subjects with breathlessness [22].

Although our data did not support an independent adverse effect of anxiety symptoms on three years incidence of CVD after adjusting for physical distress, the correlation between anxiety and physical distress opens for an underestimation of the association between anxiety and cardiovascular events. Likewise, some of the effect of physical distress on the incidence of CVD may be explained by anxiety.

Our subjects with low QOL had a tendency to have more cardiovascular events than subjects with higher QOL, adjusted for classical risk factors and HADS-anxiety. This is in line with a recent report in a similar population, where low QOL was independently associated with the risk of cerebral stroke [14], and may represent an effect common for cardiovascular diseases. However, this association was weakened when adjusted for physical distress. Although our scale measured mostly social dimensions such as social relations, the participants reported least satisfaction when asked about energy, which was an item closely related to the PSDI. Hence, one possibility is that the univariate association between QOL and cardiovascular events reflects mainly consequences of physical health, in this case by cardiovascular related symptoms.

There are several limitations to consider in interpretation and generalisation of our data. The inclusion procedures in both the Oslo Diet and Anti-Smoking Study in 1972–77 and the present DOIT may have been biased by lower prevalence of psychiatric symptoms or other individual psychosocial problems hindering motivation to participate in a lifestyle intervention study. We believe that this selection bias does not weaken our conclusion, since inclusion of subjects with more anxiety and depression at baseline would have led to a higher incidence of CVD. The significant proportion of patients with missing data could be another selection bias. As the main cause of missing data was random administration failure of questionnaires (n = 55) and the incidence of new cardiovascular events was similar to those completing the questionnaires, we believe that this bias would not have major influence on our main findings. Although our population primarily consisted of high-risk individuals without prior manifestations of CVD, a minority had verified previous cardiovascular events. However, the presence of previous events was not significantly associated with the incidence of new events during the study. In addition, our main findings were similar when considering only patients without previous CVD (data not shown). Finally, due to a limited sample size and the observational design, unknown confounders could have weakened the association between physical distress and cardiovascular events.

The clinical relevance from our results is that evaluation of subjective health complaints may contribute with valuable information in addition to standard risk evaluation in patients at high risk of CVD. Information of physical distress and quality of life can easily be obtained by all health personnel by asking a few questions of the degree of breathlessness, fatigue or peripheral coldness, or with predefined questionnaires. Such factors may be considered as elements in future prospective studies on cardiovascular health, to further evaluate this association.

## Conclusion

We have shown that increased self-reported physical distress, but not quality of life, was significantly associated with the three years incidence of cardiovascular events after adjustment for classical risk factors in an observational study of elderly men at high risk.

## Competing interests

The authors declare that they have no competing interests.

## Authors' contributions

All authors participated in drafting of the manuscript. GE: Performed the analysis and interpretation of data. ØE participated in planning the design and interpretation of data. TOK participated in planning of the design. LS participated in data analyses and interpretation. EMH conceived the idea of the study, planned the design, collected data, and participated in interpretation of the data.

## Pre-publication history

The pre-publication history for this paper can be accessed here:



## Supplementary Material

Additional file 1 **Association between physical distress, quality of life and three years incidence of cardiovascular events**. Cox proportional hazard regression model. Max and minimum scores in each tertile of Physical Symptom Distress Index: 1^st ^13–15, 2^nd ^16–20, 3^rd ^21–38; Life Satisfaction Index: 1^st ^14–22, 2^nd ^23–26, 3^rd ^27–44. HR, Hazard ratio; CI, Confidence interval. Click here for file
