# Supplementary material for: Physical distress is associated with cardiovascular events in a high risk population of elderly men
Source: BMC Cardiovasc Disord. 2009 Mar 30;9:14. doi: 10.1186/1471-2261-9-14 (PMC2667171; doi:10.1186/1471-2261-9-14)
Supplement: Additional file 1 — Association between physical distress, quality of life and three years incidence of cardiovascular events. Cox proportional hazard regression model. Max and minimum scores in each tertile of Physical Symptom Distress Index: 1st 13–15, 2nd 16–20, 3rd 21–38; Life Satisfaction Index: 1st 14–22, 2nd 23–26, 3rd 27–44. HR, Hazard ratio; CI, Confidence interval. [file 1471-2261-9-14-S1.doc]

Parameter Univariate Multivariate

HR 95 % CI p HR 95% CI p

Age 1.02 0.94 – 1.10 0.672 1.18 0.89 – 1.57 0.258

Previous cardiovascular disease 1.15 0.91 – 2.46 0.11

Previous diabetes mellitus 2.11 1.08 – 4.13 0.029

Treated hypertension 1.01 0.59 – 1.71 0.976

Current smoking 1.79 1.11 – 2.89 0.016 2.01 1.13 – 3.60 0.018

Systolic blood pressure 1.39 1.11 – 1.74 0.005 1.55 1.17 – 2.04 0.002

Pulse rate 1.06 0.83 – 1.35 0.653

Body mass index 1.06 0.83 – 1.34 0.666

Total cholesterol 1.16 0.92 – 1.46 0.214

HDL-cholesterol 0.94 0.74 – 1.20 0.641

LDL-cholesterol 1.17 0.93 – 1.49 0.188

Glucose 1.28 1.10 – 1.50 0.002 1.40 1.16 – 1.70 0.001

HADS-Anxiety

2^nd^ versus 1^st^ tertile 2.90 1.15 – 7.31 0.024

3^rd^ versus 1^st^ tertile 3.17 1.28 – 7.82 0.012

HADS-Depression

2^nd^ versus 1^st^ tertile 0.80 0.38 – 1.69 0.573

3^rd^ versus 1^st^ tertile 1.31 0.64 – 2.67 0.461

Physical Symptom Distress Index

2^nd^ versus 1^st^ tertile 2.36 0.98 – 5.70 0.056 2.10 0.87 – 5.10 0.099

3^rd^ versus 1^st^ tertile 3.72 1.60 – 8.59 0.002 2.82 1.18 – 6.75 0.020

Life Satisfaction Index

2^nd^ versus 1^st^ tertile 1.42 0.67 – 3.00 0.360 1.27 0.58 – 2.78 0.557

3^rd^ versus 1^st^ tertile 2.06 1.01 – 4.18 0.045 1.45 0.82 – 3.73 0.149
